# Rotational friction of dipolar colloids measured by driven torsional oscillations

**DOI:** 10.1038/srep34193

**Published:** 2016-09-29

**Authors:** Gabi Steinbach, Sibylle Gemming, Artur Erbe

**Affiliations:** 1 Institute of Physics, Technische Universität Chemnitz, 09107 Chemnitz, Germany; 2 Helmholtz-Zentrum Dresden-Rossendorf, Bautzner Landstrasse 400, 01328 Dresden, Germany

## Abstract

Despite its prominent role in the dynamics of soft materials, rotational friction remains a quantity that is difficult to determine for many micron-sized objects. Here, we demonstrate how the Stokes coefficient of rotational friction can be obtained from the driven torsional oscillations of single particles in a highly viscous environment. The idea is that the oscillation amplitude of a dipolar particle under combined static and oscillating fields provides a measure for the Stokes friction. From numerical studies we derive a semi-empirical analytic expression for the amplitude of the oscillation, which cannot be calculated analytically from the equation of motion. We additionally demonstrate that this expression can be used to experimentally determine the rotational friction coefficient of single particles. Here, we record the amplitudes of a field-driven dipolar Janus microsphere with optical microscopy. The presented method distinguishes itself in its experimental and conceptual simplicity. The magnetic torque leaves the local environment unchanged, which contrasts with other approaches where, for example, additional mechanical (frictional) or thermal contributions have to be regarded.

Frictional forces play a fundamental role in the dynamics of mesoscopic objects, which are omnipresent in microfluidic and biological systems. The frictional interaction of moving objects with the viscous environment affects e.g. transport, demixing, diffusion, and propulsion^1^,^2^,^3^,^4^,^5^. The study of friction during these processes also gives access to the rheological and the aggregation behavior of colloids such as ferrofluids and gels^6^,^7^,^8^,^9^. In incompressible fluids, the key quantity is the Stokes friction coefficient *f*, which is the proportionality factor between the particle velocity and the drag force. Often, many-body effects in dense systems can be expressed as an expansion of the single-particle friction. An explicit, simple equation of *f* exists for single spheres in an ideally homogeneous medium by the Stokes law. Real systems, however, face deviations from that, caused either by a non-spherical object shape^10^,^11^,^12^,^13^,^14^,^15^ or by anisotropic environments such as in non-ideal fluids or at surfaces/interfaces^16^,^17^,^18^,^19^,^20^, e.g. in a cell or a microchannel^21^,^22^,^23^. As most of these problems cannot be solved analytically one relies on approximate solutions^24^ or on experimental measurements. Friction from translational motion behaves differently from the friction during rotational motion^16^,^25^,^26^; for analysis sometimes both need to be decoupled^27^,^28^. Experimentally, the translational friction, *f*_t_, can be simply derived from the spatial displacement of a diffusing particle via optical methods. Rotational friction, *f*_*r*_, however, remains difficult to determine since additionally the orientation of the object has to be determined, which requires optical or shape anisotropy.

For nanoscopic objects, measurement methods rely on, e.g., scattering techniques^13^,^29^,^30^, time-resolved phosphorescence spectroscopy^30^, or internal reflection fluorescence microscopy^31^. In contrast, the study of microscopic objects is mainly limited to optical microscopy. One option is to measure the rotational diffusion with confocal microscopy^10^,^14^,^32^ or holographic video microscopy^11^,^33^, and then compute the friction via the Einstein–Smoluchowski relation. Another option is the application of a torque of known intensity by laser tweezers^16^,^34^,^35^, or by mechanical micro-levers^36^. From the phase lag between rotor and drive one can derive *f*_r_. While all these methods provide access to the rotational friction, a general drawback is that besides the technical expense they impose specific demands on the studied objects. For example, fluorescence labeling and a high resolution for diffusion measurements are required. When using optically or mechanically induced torques, the read-out of the phase difference between the drive and the rotator requires specific techniques, and moreover one has to correct for additional thermal or mechanical terms, which locally alter the friction coefficient.

Here, we present a technically simple approach for measuring *f*_r_ of a single particle via light microscopy using oscillating magnetic torques. We drive dipolar particles with an oscillating field, which gives a time-dependent torsional oscillation angle *θ* (*t*) of the particles. The angular amplitude *θ*_*A*_ serves as the measurable quantity^37^,^38^. Experimentally, *θ*_*A*_ can be obtained, for example, with transmission light microscopy for particles with optical inhomogeneity^39^,^40^,^41^. As an advantage of this approach, the oscillation amplitude can be experimentally extracted easily from light microscopy even at low frame rates. Additionally, magnetic fields do not influence the local friction coefficient. The main difficulty of this approach is that the torsional motion can be expressed analytically only under uniaxial fields. In that case the particles, however, do not oscillate under well defined boundary conditions. We will, therefore, first explain this problem in uniaxial fields. Then, we demonstrate theoretically how the rotational Stokes friction coefficient of single dipolar microspheres can still be obtained from the amplitude of their torsional oscillation if they are driven by biaxial homogeneous fields. Based on numerical simulations, we discuss the relation between the oscillation amplitude and the friction coefficient, and derive an approximate solution *θ*_*A*_ (*f*_r_) for the oscillation amplitude under biaxial fields. Finally, we demonstrate the applicability of this explicit equation in experimental studies. We determine the friction coefficient of single field-driven dipolar Janus microspheres from microscopy recordings.

## Results

### Uniaxial vs. biaxial field

In a highly viscous environment, where inertia can be neglected, the equation of rotation of a particle with magnetic moment **m** exposed to an external field **B** is obtained by balancing the magnetic torque, *τ*_*m*_ = **m** × **B**, against the viscous drag, 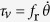, with the angular velocity 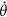 of the particle.

**Figure 1:**
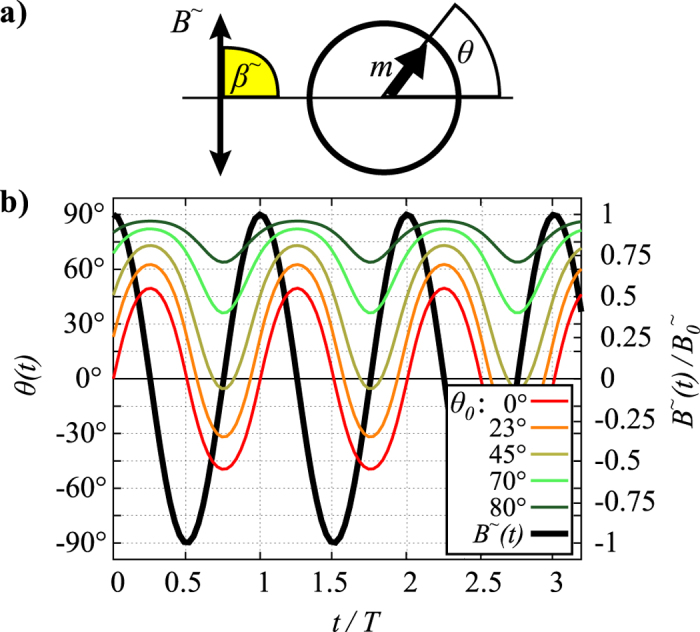
Oscillations in a uniaxial field. (**a**) Sketch of the dipolar particle with magnetic moment *m* in an oscillating field *B*^∼^. (**b**) Analytic functions of the angular oscillation *θ* (*t*) of a dipolar particle under a field *B*^∼^ according to equation (2) for different starting values *θ*_0_, and the function of the applied field *B*^∼^ (*t*).

We first consider a dipolar particle driven by a uniaxial, oscillating field 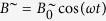 (Fig. 1a). We assume that the particle takes orientations *θ* in the value range [−90°, …, 90°] and that the field points along a radial field angle of *β*^∼^ = 90°. In this case, the equation of rotational motion of is given by


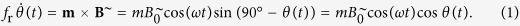


This differential equation has an analytic solution given by


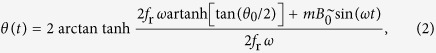


where *θ*_0_ is the initial value of the radial orientation of the particle. *θ*_0_ has no influence on the phase lag, which is exactly *π*/2, but *θ*_0_ crucially impacts the form and the amplitude of the oscillation in a uniaxial, oscillating field (Fig. 1b). The dipolar particle performs a symmetric oscillation with respect to the ordinate only if the field is applied perpendicular to the initial orientation of the dipole. Here, this corresponds to *β*^∼^ = 90° and *θ*_0_ = 0°. The curve becomes asymmetric if *θ*_0_ ≠ 0°. The form of the oscillation is, thus, strongly determined by *θ*_0_. For a distribution of particles with random orientation *θ*_0_ under a uniaxial, oscillating field, the amplitudes *θ*_*A*_ are, thus, not a distinct measure for the frictional drag. In a real system of colloidal particles additionally thermal fluctuations render an analysis difficult, because the fluctuations effectively alter *θ*_0_ continuously. The fluctuation is most prominent when *B*^∼^ crosses zero during the oscillation, that is at low magnetic coupling parameters Γ, defined as the ratio between the magnetic interaction and the thermal energy.

**Figure 2:**
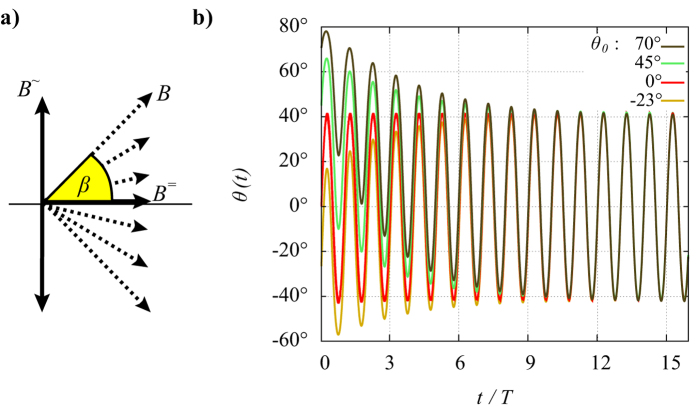
Oscillations in a biaxial field. (**a**) Sketch of the field vector *B* of a combined oscillating field *B*^∼^ and static field *B*^=^. (**b**) Numerically calculated transient oscillation *θ* (*t*) of a dipolar particle in the biaxial field (*B*^∼^, *B*^=^) for different starting values *θ*_0_.

To quantitatively study the driven oscillation of single particles under well-defined boundary conditions, one has to ensure uniform oscillations and thermal fluctuations have to be suppressed by sufficiently large Γ values at all times. This can be realized if an oscillating magnetic field 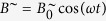 is superimposed by a perpendicular static field *B*^=^ (Fig. 2a). Note that here the orientation of the field changes periodically. The maximum field angle is 
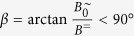
. On a dipolar particle, *B*^∼^ causes a radial oscillation *θ* (*t*) and *B*^=^ exerts a permanent aligning torque that pulls the particle back to *θ* = 0°. We assume the thermal energy to be sufficiently low if Γ > 1000, that is if 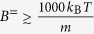. Under such a biaxial field, the torsional oscillation of a single particle is given by


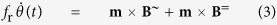



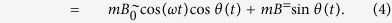


Unfortunately, there exists no analytic solution *θ* (*t*) for such a differential equation. From numerical calculations we have found that *B*^=^ causes the dipole to always approach a symmetric steady-state oscillation through a transient oscillation (Fig. 2b). Then, the steady-state oscillation amplitude *θ*_*A*_ becomes independent of the actual initial orientation *θ*_0_. For a particle with parameters *m* and *f*_r_, the amplitude *θ*_*A*_ is uniquely defined for given values *B*^=^, *B*^∼^, *ω* of the biaxial field. For that matter, *θ*_*A*_ in such a biaxial field can be used as a measure for the remaining system parameters *m* or *f*_r_ in equation (4). The only drawback in this approach is that there does not exist an analytic relationship between these system parameters and *θ*_*A*_ since equation (4) is not solvable. Instead, we derive an approximate function as presented below.

### Oscillation amplitudes under biaxial fields

The oscillation amplitude *θ*_*A*_ depends on the interplay between the interaction of the dipole with the magnetic field and the frictional drag of the particle in the viscous medium. Assuming large values of 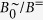 such that 
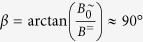
, the steady-state oscillation of a single particle can be approximated by the analytic solution, equation (2), of the oscillation in a uniaxial oscillating field (*B*^=^ = 0). In this case, the oscillation amplitude *θ*_*A*_ is given by


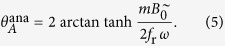


**Figure 3:**
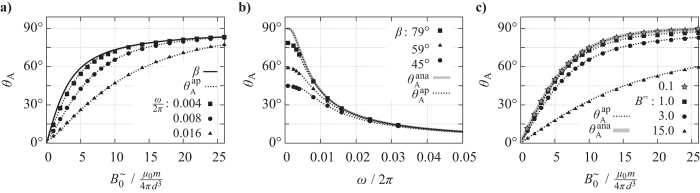
Numerical study of the oscillation amplitude *θ*_*A*_ as a function of the field parameters *B*^=^, 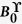, *ω*. Filled symbols correspond to numerically obtained points obtained for an ideal dipole with (**a**) 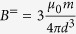, (**b**) 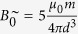, (**c**) *ω*/2*π* = 0.008. Grey solid lines give the analytic curve 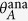 according to equation (5) and dotted lines correspond to approximate curves 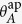 according to equation (6) where *B*^=^ ≠ 0.

In the following, the impact of the field parameters *B*^=^, 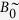, *ω* on the oscillation amplitude *θ*_*A*_ of an ideal dipolar sphere and associated deviations from the analytic solution 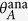, equation (5), for *β* ≠ 90° will be explained based on numerical simulations. For simplicity, we assume that *T* = 0 and do not incorporate thermal fluctuations in the simulations. The results are presented in Fig. 3a–c, where filled symbols correspond to numerically determined data points.

First, *θ*_*A*_ is presented as a function of the field amplitude 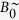 (Fig. 3a). With increasing 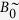 one can see that *θ*_*A*_ asymptotically approaches 90°. (The final convergence is not visible within the value range plotted in Fig. 3a, where the graphs reach only 85° at maximum.) The convergence is slowed down by increasing values of *ω*. In the case of vanishing frequency the curve reaches the limiting function 
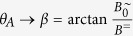
, indicated as solid line in Fig. 3a. Next, the functional dependence of *θ*_*A*_ on *ω* is shown in Fig. 3b. Here, the field angle *β* is varied among the different data sets. In accordance with the trend in Fig. 3a, the amplitude *θ*_*A*_ decreases with increasing *ω* and converges to 0°. In the limit of *ω* = 0, there is no oscillation and, thus, *θ*_*A*_ (*ω* = 0) = *β* must hold. The variation of *β* results in a vertical compression or stretching of the curves *θ*_*A*_ (*ω*). In the limit of *β* = 90°, which corresponds to vanishing *B*^=^, the data points approach an upper limit given by the analytic solution 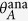. Finally, the dependence 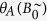 is examined again, but now *B*^=^ > 0 is varied among the data sets while *ω* is kept constant (Fig. 3c). Again, *θ*_*A*_ increases with 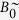 and asymptotically approaches 90°. The convergence becomes steeper if *B*^=^ decreases. For the smallest value examined here 
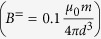
 the data points almost coincide with the analytic solution 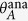 in the absence of *B*^=^, as indicated by the solid, grey curve. This proves that equation (5) provides a good approximation, assuming small values of *B*^=^/*B*^∼^.

The analysis of the trends and of the boundary values of *θ*_*A*_ upon varying *B*^=^, *B*^∼^ and *ω* provides suitable reference points for the search of a unique, approximate function 
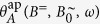
. Technically, the search must be based on the simulation data for which both system parameters, the magnetic moment *m* and the rotational friction coefficient *f*_r_, are predefined input values. The detailed derivation of a suitable approximate function 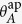 is provided in the Supplementary Information in detail and will be summarized here only shortly. The key boundary conditions are that 
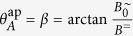
 must hold for vanishing *ω* and that equation (5) holds for vanishing *B*^=^. An approximate expression can be obtained from a multiplication of both. Including some necessary correction factors we suggest that


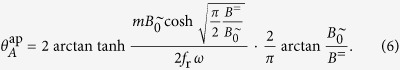


Correction factors are indicated by red letters. The normalization factor 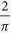 accounts for the fact that the multiplication of equation (5) with arctan 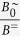 leads to a value range of 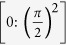 but it must hold that 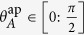. An additional adjustment term cosh[..] has been determined phenomenologically (Supplementary Information) to account for small deviations in the range of large *β* (Fig. S2, Table S1). Plotting 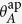 for the respective field parameters and the values *m* = 1 and 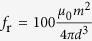 used in the simulation (see Numerical Methods) shows a consistent agreement between the approximate function and the numerically obtained data sets (Fig. 3a–c). This indicates that equation (6) is a suitable approximation for the oscillation amplitude of a dipole in the applied biaxial field. This means in turn that from the measurement of *θ*_*A*_ under applied field values *B*^=^, 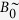, *ω* one can derive either dipole moment *m* or the Stokes friction coefficient *f*_r_ using equation (6).

### Oscillation amplitudes of driven Janus colloids

**Figure 4:**
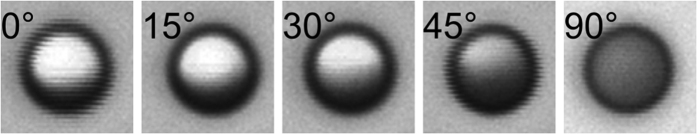
Microscopy images of a magnetic Janus particle with a diameter of 4.54 *μ*m for different orientations *θ*. The ratio between the projected area of the transparent (bright) hemisphere and the coated (black) hemisphere serves as measure for the radial orientation *θ* of the particle.

Silica particles with a hemispherical magnetic coating, called Janus particles, enable the experimental study of rotational motion via transmission light microscopy^41^,^42^. The Janus director, which corresponds to the rotational symmetry axis of such particles, is visualized by the optical inhomogeneity between the coated and the uncoated hemispheres (Fig. 4). In the system studied here, the net magnetic moment of the particles points along the Janus director^39^. In this case the magnetic orientation of a particle coincides with the orientation of the optical contrast. We investigate particles with a diameter of *d* = 4.54 *μ*m after sedimentation in water on a substrate glass. The applied oscillating field *B*^∼^ points perpendicular to the substrate plane. Then the particles perform driven oscillations around an axis parallel to that plane. For such a radial oscillation, the orientation of a particle is obtained from the area ratio between the projection of the uncoated (bright) and the coated (black) hemisphere (Fig. 4). Note that due to the projection only the absolute value of *θ*_*A*_ can be extracted. The image analysis is limited to amplitudes 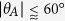 for the optical reasons of diffraction and total reflection.

The studied particles have a mean dipole moment of *m* = 6.4 · 10^9^ *μ*_B_, which we have determined by SQUID magnetometry of an array of coated particles. In a constant field of *B*^=^ ≈ 0.01 mT, they have a magnetostatic energy of *mB*^=^ ≈ 1440 *k*_B_*T*, and, thus, thermal fluctuations are suppressed sufficiently at all times. Consequently, in combined fields *B*^=^ and 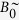, the non-interacting Janus particles oscillate uniformly after a transient time, and *θ*_*A*_ can be measured under well-defined conditions.

**Figure 5:**
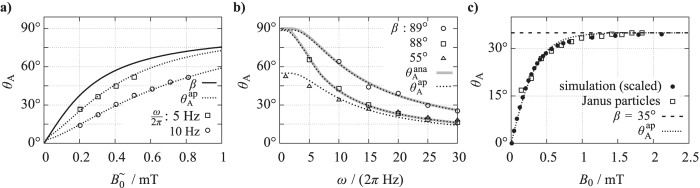
Experimental study of the oscillation amplitude *θ*_*A*_ as a function of the field parameters *B*^=^, 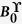, *ω*. Open symbols correspond to data points obtained experimentally for the Janus particles with (**a**) *B*^=^ = 0.260 mT, (**b**) for *β* = 89° (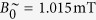, *B*^=^ = 0.017 mT); 88° (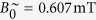, *B*^=^ = 0.017 mT); 55° (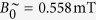, *B*^=^ = 0.390 mT), (**c**) *ω*/2*π* = 5 Hz. Grey solid lines give the analytic curve 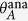 given by equation (5) and dotted lines correspond to approximate curves 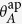 given by equation (6).

The data points obtained from measurements of Janus particles (Fig. 5) qualitatively show the same tendencies of *θ*_*A*_ as an ideal, dipolar sphere. Figure 5a shows that *θ*_*A*_ is an increasing function of 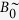. An increase of *ω* results in a less steep increase of *θ*_*A*_. This is proven explicitly by the trend that *θ*_*A*_ (*ω*) decreases with increasing *ω* as shown in Fig. 5b for three different sets of field intensities. Experimental data sets with *β* ≈ 90° can be approximated by 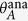, as well. Besides *β* and *ω*, also the maximum field intensity 
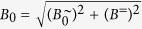
 affects *θ*_*A*_. Exemplarily, we have measured *θ*_*A*_ for a set of field intensities *B*_0_ with the same maximum field angle of *β* = 35° (Fig. 5c). The experimental data points (unfilled symbols) show that *θ*_*A*_ converges to *β* with increasing *B*_0_.

While the trends in the experimental (Fig. 5) and the numerical (Fig. 3) data compare well qualitatively, a quantitative comparison is not straightforward, because numerical time steps are not an equivalent of the real time. Consequently, the numerical and experimental values for the field frequency *ω* cannot be related to each other. At the end of this section we will show that a comparison is, however, possible if the experimental friction coefficient *f*_r_ is known, which will be determined next.

The coincidence in the trends of the experimental and the simulation data suggests that the model of a dipolar sphere reproduces the dynamics of single ferromagnetic particles. Therefore, equation (6) applies to the oscillation amplitude of the Janus particles also. Using this approximate function 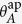 we can extract the Stokes friction coefficient by fitting the experimental data sets in Fig. 5 via *f*_r_. As noted already above, the friction of a particle rotating around an axis parallel to the substrate plane is examined here. The fit values *f*_r_ of the individual data sets with 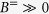 are listed in Table 1 together with the applied field parameter values. The mean value amounts to 
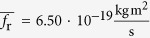
 with a maximum deviation of 5% from that mean value. Using the fit values *f*_r_ and the given field values, the corresponding graphs 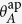 are plotted in Fig. 5. The curves show very good agreement with the trend of the experimental data. For comparison, for data sets with very small *B*^=^ the curve 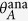 has been plotted in addition (Fig. 5b, grey solid lines). Apart from the range of very small *ω*, these curves coincide surprisingly well with those of 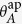. This proves that for very small *B*^=^ also the experimentally obtained amplitude *θ*_*A*_ is fairly well approximated by 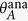.

The major source of error in this approach arises from the determination of the oscillation amplitude from the black-white ratio in the projected images of the particles. The measured ratio is very sensitive to the focus adjustment of the optical microscope. Deviations in the focus height from the one at which the calibration curve has been recorded directly cause an error of *θ*_*A*_. From a number of reference measurements with seven particles each, we have determined an error of Δ*θ*_*A*_ = ±4° that is achievable within the adjustment accuracy of our setup. The relative error of the friction value *f*_r_ for each data point (*θ*_*A*_, *B*^=^, *B*^∼^, *ω*) in Fig. 5 is then given by 
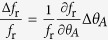
, where the function *f*_r_ is obtained from solving equation (6) for *f*_r_. This amounts to a maximum value of 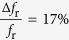 for the data sets presented here.

Finally, the quantitative comparison between experiment and simulation can be conducted. The experimental field intensity, measured in mT, can be converted to the numerical unit 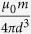 of the field intensity by 
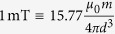
 with the experimental values of *m, d*. As mentioned above, the full comparability requires a reference value for the numerical time steps. This can be obtained from the friction coefficient. While the real friction coefficient is measured in 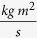, the numerical value has the dimension of energy, 
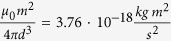
. Thus, the ratio of both has the dimension of time in *s*. With the value used in the simulation, 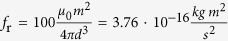, and the experimental mean value obtained from fitting, 
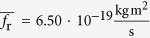
, this gives a numerical time step of Δ*t* = 1.73 · 10^−3^ *s*. An experimental frequency of *ω*/2*π* = 5 Hz, thus, corresponds to a numerical frequency of *ω*/2*π* = 0.008. Using this frequency and taking the field conversion into account, a quantitative comparison is possible. As an example, numerical data sets *θ*_*A*_(*B*_0_) of an ideal dipole for the case *β* = 35° have been calculated and drawn in Fig. 5c (filled symbols). The agreement with the experimental data proves that the ideal dipolar particle is also a suitable model for the quantitative analysis of single dipolar Janus particles.

## Discussion

Here, we have presented a measurement tool for the rotational friction of single dipolar colloids. We have demonstrated that the friction coefficient can be obtained from the amplitude of driven torsional oscillations of the particles under biaxial magnetic fields. A semi-empirical equation has been derived that relates the oscillation amplitude with the friction coefficient. The validity of this equation has been tested experimentally with dipolar Janus spheres. For such particles with a diameter of *d* = 4.54 *μ*m in water on a glass substrate we have determined a value of 
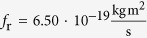
.

From such a measurement one can gain further insight into the studied system. Here, the distance between particle and substrate is accessible. According to the Stokes equation, *f*_r_ = *πηd*^3^, ideally such a sphere in a homogeneous aqueous environment (*η* = 1 mPa s) has a rotational friction coefficient of 
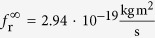
. The overestimation of the experimentally obtained value of *f*_r_ for the Janus particles is caused by their proximity to the substrate. The effect of the wall-particle distance on the rotational friction has been detailed in many previous studies^16^,^18^,^19^,^20^,^25^,^36^. According to the solution given by *Dean* and *O*’*Neill*^43^, here, the wall-particle distance is approximately 0.01 *d*, which is 0.05 *μ*m. An additional, but small source for the difference between *f*_*r*_ and 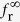 is given by the fact that the magnetic center is shifted radially away from the particle center^39^ since the coating covers only one hemisphere. Since an externally applied torque acts on the magnetic center, the particle will rotate around a non-centric axis. This results in an increased effective hydrodynamic radius 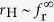 of the particles. In the presented experiment, this effect can, however not be separated from the influence of the surface. Based on the recorded videos we, however, suggest that this increase in *r*_H_ is very small since we could not measure it. Here, the influence of the non-spherical shape of the particle due to the coating can be neglected since the coating has a maximum height of less than 0.2%*d*.

A macroscopic equivalent of the discussed torsional oscillation of a sphere has been presented earlier^37^ using cm-sized ferromagnetic spheres in a highly viscous fluid exposed to an oscillating magnetic field. There, the constant aligning torque has been realized by gravitational forces that act on a sphere with non-symmetric mass distribution. The authors reported similar qualitative behavior of the oscillation amplitude as we have discussed here. Therefore, we suggest that the concept of an approximate function for *θ*_*A*_ can be applied for a quantitative analysis in other systems. If gravitational forces are applied, then the magnetic moment and the torque-exerting field have to be replaced by the mass and the gravitational force accordingly.

In summary, the approximate equation 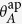 for the oscillation amplitude of a particle driven by superimposed oscillating and static fields provides an alternative, reliable method to measure the rotational Stokes friction coefficient *f*_r_ (or alternatively the net magnetic moment) of micron-sized Janus particles in a straightforward and experimentally cheap way. The advantage of this method is that the application of a magnetic torque does not alter the frictional interaction between the object and the environment, and no corrections for additional energy contributions are necessary. Experimentally, the determination of the oscillation amplitude is the major source of measurement error, transferring into a relative error of 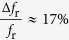 for the studied Janus particles. This error could, however, be considerably smaller for other particles for which the oscillation can be read out with higher precision.

The experimental study presented here discusses only spherical particles. This is, however, not a general limitation as the numerical study applies for any dipolar object, e.g., also ellipsoids^44^,^45^,^46^ etc. Therefore, this method provides a promising tool in the growing subject of anisotropic magnetic particles in soft materials^47^. The effect of shape anisotropy of dipolar particles^48^,^49^,^50^,^51^ on the rotational friction could be measured with the presented method. In addition, initially non-magnetic objects potentially can be studied after magnetic functionalization using recently promoted synthesis techniques based on microfluidics^52^,^53^ or surface modification^40^,^54^,^55^, which has been applied here^56^. In future, the presented method may provide a basis for the study of rotational friction in many-particle systems. From the difference between the oscillation amplitudes of single particles and of particles in dense suspension one could extract a quantitative analysis of the particle interaction. This concerns the change of the friction coefficient due to the modification of the hydrodynamic drag in the proximity to other particles, and the explicit interparticle interaction, e.g., the magnetic coupling. This promises an additional way to quantitatively analyze the numerously reported non-equilibrium systems of microscopic magnets such as chains, rods and filaments under time-dependent fields^39^,^45^,^46^,^57^,^58^,^59^,^60^.

## Methods

### Numerical Methods

The rotational motion of a sphere with dipole moment *m*, that is exposed to a biaxial, orthogonal field, consisting of an oscillating field 
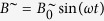
 and a static field *B*^=^, has been calculated by numerically solving the equation of rotation (equation (4)). In this simulation, the particle is spatially fixed at its center. The orientation *θ*_*t*+1_ of the sphere at time step *t* + 1 is obtained iteratively by


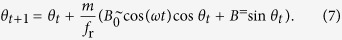


In the calculation, the magnetic moment is set to *m* = 1. A rotational friction coefficient of 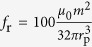 has been applied. It has the dimension of energy since the numerical time steps Δ*t* = (*t* + 1) − *t* are dimensionless and set to 1. The angular frequency *ω* is also a dimensionless quantity.

### Experimental Details

The particle preparation and experimental setup has been described in detail previously^39^. In short, silica spheres with a diameter of *d* = (4.54 ± 0.45) *μ*m have been coated on one hemisphere with a magnetic thin film according to an established recipe^56^. The coating consists a multilayer stack of Ta(3.0 nm)/Pd(3.0 nm) [Co(0.28 nm)/Pd(0.9 nm)]_8_/Pd(1.1 nm). This stacking is known to exhibit strong magnetic anisotropy where the easy axis points perpendicular to the film surface^61^. When depositing such a film on a spherical particle, the anisotropy axis points perpendicular to the particle surface. After magnetic saturation, the film becomes single domain^62^, which results in a radially symmetric anisotropy orientation across the particle surface^56^. This anisotropy distribution has been confirmed also theoretically^63^. Such a particle exhibits a stray field with dipolar characteristic, and the net dipole moment points perpendicular to the magnetic cap. A dilute suspension of such magnetically capped particles in distilled water is studied via transmission light microscopy. Following the density mismatch, the particles sediment on the bottom of the sample cell, which had been treated by plasma cleaning prior to sample preparation. To optimize the recording quality for digital image analysis, the microscopy illumination has been adjusted for homogeneous illumination via a condenser lense, and the sample has been aligned perpendicular to the light path using an adjustable stage. In addition, we have used a Lica objective (HC PL FL L 63x/0.70 CORR PH2) with an adjustable collar to correct for spherical aberration artifacts caused by the presence of the substrate glass. The attached digital camera (Leica DFC295) has a video resolution of 864 px × 648 px. An electromagnetic coil is mounted above the sample cell, providing perpendicular low-frequency fields. An additional set of two pairs of coils attached beneath the sample provide constant fields parallel to the sample plane. The microscopy recordings of single particles have been analyzed by optical image analysis using the open-source software ImageJ and the included plugin ‘particle analysis’. After converting the recordings into black-white threshold bitmaps, the ImageJ plugin measures the projected area of the transparent hemisphere of a particle over several oscillation cycles. Due to the stroboscopic microscopy recording with low frame rate (20 fps), one obtains an overlaid beat of the oscillating curve area vs. time. The minimum value of the area is extracted and, using a calibration curve, this value is correlated with an oscillation amplitude *θ*_*A*_.

## Additional Information

**How to cite this article**: Steinbach, G. *et al*. Rotational friction of dipolar colloids measured by driven torsional oscillations. *Sci. Rep.* **6**, 34193; doi: 10.1038/srep34193 (2016).

## Supplementary Material

Supplementary Information

## Figures and Tables

**Table 1:** Values of the rotational friction coefficient *f*_r_ obtained from fitting the experimental data sets (Fig. 5a–c) of different dependencies (2nd column) to 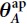 or 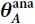, respectively, using the applied field parameters (3rd column).

Figure 5 panel	Function	Field parameters			*f*_r_ 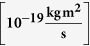
(a)	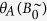	*B*^=^ = 0.260 mT	*ω*/2*π* = 5 Hz		6.49
		*B*^=^ = 0.260 mT	*ω*/2*π* = 10 Hz		6.34
(b)	*θ*_*A*_ (*ω*_*B*_)	*B*^=^ = 0.390 mT	*B*^∼^ = 0.558 mT	(*β* = 55°)	6.26
		*B*^=^ = 0.017 mT	*B*^∼^ = 1.015 mT	(*β* = 89°)	6.85
		*B*^=^ = 0.017 mT	*B*^∼^ = 0.607 mT	(*β* = 88°)	6.81
(c)	*θ*_*A*_ (*B*_0_)	*β* = 35°	*ω*/2*π* = 5 Hz		6.27
