# Supplementary material for: Rotational friction of dipolar colloids measured by driven torsional oscillations
Source: Sci Rep. 2016 Sep 29;6:34193. doi: 10.1038/srep34193 (PMC5040963; doi:10.1038/srep34193)
Supplement: Supplementary Information [file srep34193-s1.pdf]

Supplementary information: Rotational friction of dipolar colloids measured by driven torsional oscillations

Gabi Steinbach[1,2], Sibylle Gemming[2], and Artur Erbe[1,2]

[1]Institute of Physics, Technische Universität Chemnitz, 09107 Chemnitz, Germany.
[2]Helmholtz-Zentrum Dresden-Rossendorf, Bautzner Landstrasse 400, 01328 Dresden, Germany.

Phenomenological determination of an approximate solution for θ_A

The torsional oscillation of a dipolar particle driven by a magnetic field consisting of two orthogonal components, an oscillating one ($B^{\sim} = B_0^{\sim}\cos(\omega t)$) and a static one ($B^{=}$), is described by the differential equation

$$
\begin{aligned}
f_{\mathrm{r}}\dot{\theta}(t) &= \mathbf{m}\times\mathbf{B}^{\sim}+\mathbf{m}\times\mathbf{B}^{=} &(1)\\
&= mB_0^{\sim}\cos(\omega t)\cos\theta(t)+mB^{=}\sin\theta(t). &(2)
\end{aligned}
$$

Under such conditions, dipolar particles perform well-defined oscillations, and the study of the oscillation amplitude θ_A only is sufficient for a quantitative analysis of the rotational friction. There exists no analytic equation for θ_A, but one can derive an approximate expression as follows.

θ_A depends on three field parameters, $B_0^{\sim}$, ω, $B^{=}$, and two fixed system parameters, the rotational friction coefficient f_{r} and the magnetic moment m of a particle. A general understanding of the dependence of θ_A on the field parameters can be obtained from a boundary value analysis. θ_A reaches certain limiting values if one of the field parameters becomes either zero or infinite (Figure S1). For the case that $B^{=} = 0$ the rotational motion can be solved analytically. Assuming an initial dipole orientation of $\theta_0 = 0^\circ$ gives the amplitude function

$$
\theta_A^{\mathrm{ana}}(B^{=}=0) = 2\arctan\tanh\frac{mB_0^{\sim}}{2f_{\mathrm{r}}\omega}. \qquad (3)
$$

Sketching equation (3) as a function of $B_0^{\sim}$ and of ω (Figure S1 a, b) reveals that θ_A converges to $\theta_A = 90^\circ$ if $\omega \to 0$ or $B_0^{\sim} \to \infty$ and θ_A becomes 0° if $\omega \to \infty$ or $B_0^{\sim} \to 0$. Next, we consider the case of vanishing field oscillation ($\omega = 0$) while $B^{=} > 0$ is applied (Figure S1 b, c). In this case the dipole obtains a static orientation given by

$$
\theta_A^{\mathrm{st}}(\omega=0) = \beta = \arctan\frac{B_0^{\sim}}{B^{=}}. \qquad (4)
$$

The difficulty is now in finding a single equation that fulfills the boundary conditions given by equation (3) and equation (4), and that is also valid in the intermediate ranges. The most striking consequence of combining non-zero ω and $B^{=}$ (dashed curves in Figure S1 a, c) is the vertical compression of the curves given by equation (3) and by equation (4) such that θ_A decreases with respect to the cases $\theta_A(B^{=}=0)$ and $\theta_A(\omega=0)$.

In the following, an approximate function for the oscillation amplitude will determined based on phenomenological arguments. A first guess for an approximate solution θ_A^{ap} is given by simply multiplying the dynamic term (equation (3)) and the static term (equation (4)), which gives

$$
\theta_A^{\mathrm{ap1}} = 2\arctan\tanh\frac{mB_0^{\sim}}{2f_{\mathrm{r}}\omega}\,\frac{2}{\pi}\arctan\frac{B_0^{\sim}}{B^{=}}. \qquad (5)
$$

The correction by $\frac{2}{\pi}$ (red) is a normalization factor. It accounts for the fact that equation (3) and equation (4) both become $\frac{\pi}{2}$ in the case of vanishing ω or $B^{=}$, respectively, but $\theta_A \leq \frac{\pi}{2}$. To test equation (5), we compare $\theta_A^{\mathrm{ap1}}(B_0^{\sim})$ with the data points $(\theta_A^{\mathrm{ns}};B_0^{\sim})$ of a single dipolar particle, which is obtained from numerically solving (ns) equation (2). System parameter with the values $f_{\mathrm{r}} = 100\,\frac{\mu_0 m^2}{4\pi d^3}$ and $m = 1$ are used in the numerical study. The absolute difference $\theta_A^{\mathrm{ap1}} - \theta_A^{\mathrm{ns}}$ at the measurement points is provided in Table S1. The approximate function and the numerical data points are plotted in Figure S2 a,b. It shows that θ_A^{ap1} underestimates θ_A^{ns} in the low-field regime of $B_0^{\sim}$ (Figure S2 b), thus, in the range where $B^{=}$ has a considerable impact.

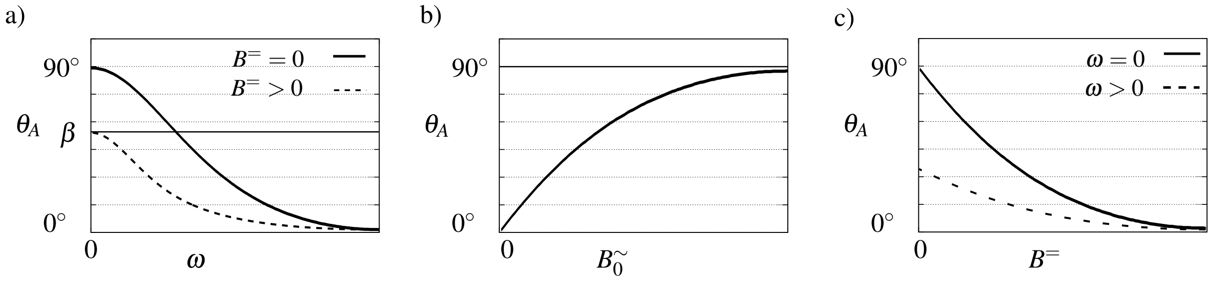

Figure S1. Sketched trends of the oscillation amplitude θ_A as a function of the field parameters **a**) ω, **b**) $B_0^{\sim}$ and **c**) $B^{=}$.

The next step of approximation is based on the logical consideration that any further correction of the oscillation amplitude should be attributed to the dynamic term arising from equation (3). The simplest form of a correction is given by incorporating a factor C such that

$$\theta_A^{\mathrm{ap1}C} = 2\arctan\tanh\frac{mB_0^{\sim}C}{2f_\mathrm{r}\omega}\,\frac{2}{\pi}\arctan\frac{B_0^{\sim}}{B^{=}}. \tag{6}$$

Theoretically, this factor C can be a function of any of the field parameters. To figure out whether one can rule out the dependence of C on one or more field parameters, the following test is performed: If C does not depend on a certain field parameter X then there should exist a constant value C by which $\theta_A^{\mathrm{ap1}C}(X)$ reproduces the numerical data $\theta_A^{\mathrm{ns}}(X)$ while the other parameter values are fixed. Exemplarily, this is shown in Figure S2 c, where $\theta_A^{\mathrm{ns}}(\omega)$ is plotted for two different sets of field intensities. By fitting both numerical data sets with respect to equation (6) via C, a fit value is obtained by which $\theta_A^{\mathrm{ap1}C}$ reproduces θ_A^{ns} fairly well over the full range of examined ω. (Of course, the fit value of C differs among both data sets since different field intensities have been used.) Such a matching between the numerical data points and the approximate equation cannot be obtained when fitting to the functions $\theta_A^{\mathrm{ap1}C}(B_0^{\sim})$ and $\theta_A^{\mathrm{ap1}C}(B^{=})$ via C. It can, thus, be concluded that C depends on $B_0^{\sim}$ and $B^{=}$ only.

Next, a suitable form of C will be determined that reduces the deviation between θ_A^{ap1} and θ_A^{ns}. To account for the limiting case $\theta_A(B^{=}=0)$ (equation (3)) it is necessary that C must become 1 if $B^{=}$ vanishes. The closer inspection of the deviation between θ_A^{ns} and θ_A^{ap1} (Table S1) suggests that C should increase if $B^{=}$ increases or if $B_0^{\sim}$ decreases. A number of mathematical functions that meet both criteria (e.g., $e^{\frac{B^{=}}{B_0^{\sim}}}$, $\cosh\frac{B^{=}}{B_0^{\sim}}$, $\frac{B^{=}}{B_0^{\sim}}+1$) have been tested. Among them, the function $C=\cosh\left(\frac{B^{=}}{B_0^{\sim}}\right)$ results in the best approximation over the whole field and frequency range. It gives the next iteration of approximation

$$\theta_A^{\mathrm{ap2}} = 2\arctan\tanh\frac{mB_0^{\sim}\cosh\frac{B^{=}}{B_0^{\sim}}}{2f_\mathrm{r}\omega}\,\frac{2}{\pi}\arctan\frac{B_0^{\sim}}{B^{=}}. \tag{7}$$

Figure S2 b and Table S1 show that θ_A^{ap2} reduces the underestimation of θ_A^{ap1} with respect to θ_A^{ns}. There emerges, however, an overestimation for very small values of $B_0^{\sim}$. Both the small over- and the underestimation are further reduced by applying the square root to $\frac{B^{=}}{B_0^{\sim}}$, which gives

$$\theta_A^{\mathrm{ap3}} = 2\arctan\tanh\frac{mB_0^{\sim}\cosh\sqrt{\frac{B^{=}}{B_0^{\sim}}}}{2f_\mathrm{r}\omega}\,\frac{2}{\pi}\arctan\frac{B_0^{\sim}}{B^{=}}. \tag{8}$$

This function still consistently underestimates θ_A^{ns} for small field amplitudes, but much less than θ_A^{ap1}. Now, an additional parameter q is introduced in the term $\cosh\sqrt{q\frac{B^{=}}{B_0^{\sim}}}$. Again, the dependence of q on the field parameters is tested using the same strategy as applied for analyzing C above. It is found that q negligibly depends on $B_0^{\sim}$ and $B^{=}$. As an approximation q can be assumed to be constant. The fit via q against several numerical data sets (Figure 2) has revealed that $q\approx\frac{\pi}{2}$. This gives the final approximate solution

$$\theta_A^{\mathrm{ap4}} = 2\arctan\tanh\frac{mB_0^{\sim}\cosh\sqrt{\frac{\pi}{2}\frac{B^{=}}{B_0^{\sim}}}}{2f_\mathrm{r}\omega}\,\frac{2}{\pi}\arctan\frac{B_0^{\sim}}{B^{=}}. \tag{9}$$

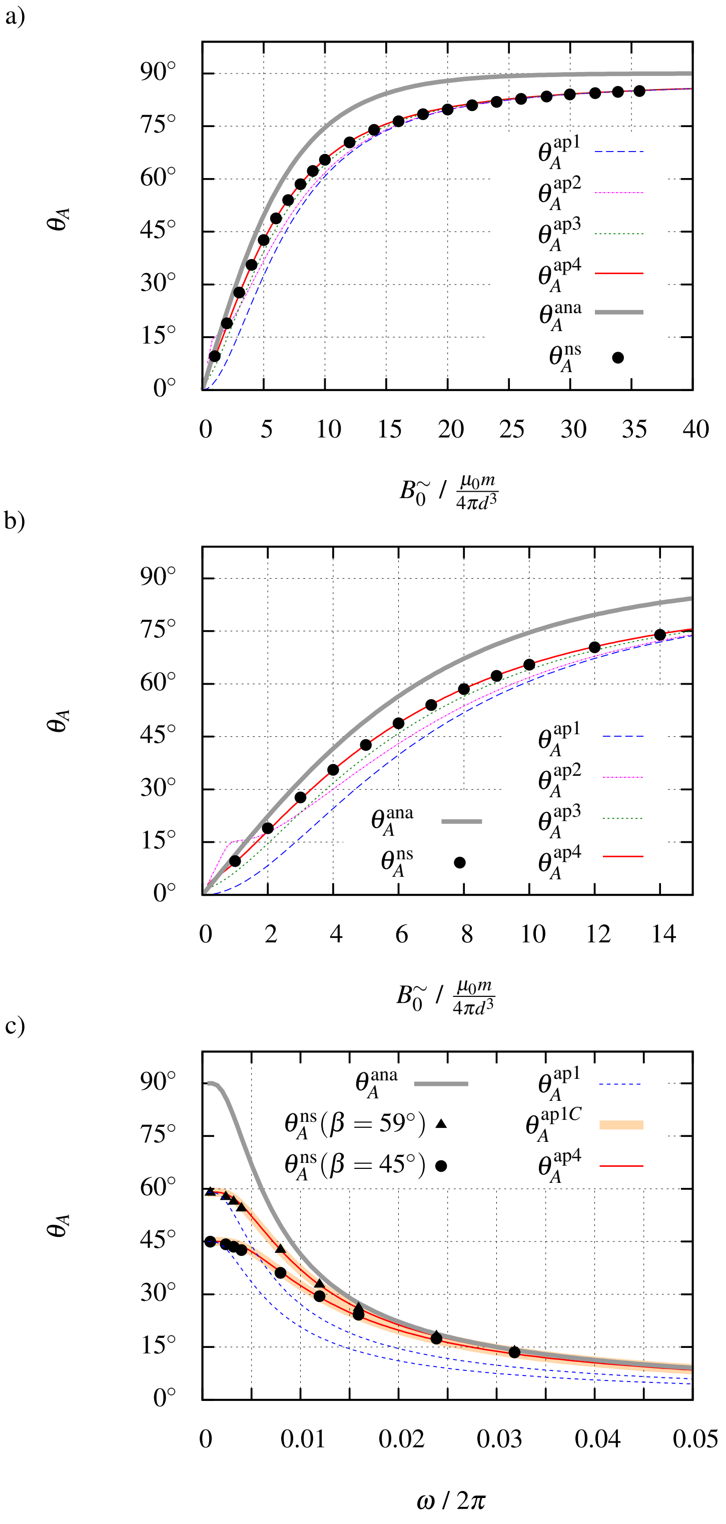

Figure S2. Numerically obtained oscillation amplitudes θ_A^{ns} (dots) of a dipolar particle and approximate functions $\theta_A^{ap(n)}$ (curves), for the step of approximation $n = 1, \ldots, 4$ (equation (5) - equation (9)), **a**) as a function of the field amplitude $B_0^{\sim}$ ($\omega = 0.05/2\pi$, $B^{=} = 3\,\frac{\mu_0 m}{4\pi d^3}$), **b**) a zoom of a) for small values of $B_0^{\sim}$, and **c**) as a function of the field frequency ω for $B^{=} = 3\,\frac{\mu_0 m}{4\pi d^3}$ ($\beta = 59°$) and for $B^{=} = 5\,\frac{\mu_0 m}{4\pi d^3}$ ($\beta = 45°$) where $B_0^{\sim} = 5\,\frac{\mu_0 m}{4\pi d^3}$.

$B_0^{\sim}$	θ_A^{ns}	$\theta_A^{ap1} - \theta_A^{ns}$	$\theta_A^{ap2} - \theta_A^{ns}$	$\theta_A^{ap3} - \theta_A^{ns}$	$\theta_A^{ap4} - \theta_A^{ns}$
1	9.61	-7.28	5.71	-3.13	-0.34
2	18.97	-10.61	-1.24	-4.38	-0.80
3	27.71	-11.47	-4.32	-4.32	-0.51
4	35.59	-11.03	-5.53	-3.81	-0.13
5	42.65	-10.11	-5.91	-3.32	0.04
6	48.76	-8.96	-5.75	-2.84	0.12
7	54.00	-7.76	-5.32	-2.40	0.15
8	58.48	-6.66	-4.80	-2.05	0.13
9	62.25	-5.61	-4.20	-1.70	0.14
10	65.44	-4.69	-3.61	-1.39	0.14
12	70.39	-3.18	-2.54	-0.88	0.19
14	73.95	-2.06	-1.69	-0.48	0.25
16	76.41	-1.15	-0.92	-0.07	0.43
18	78.38	-0.64	-0.51	0.09	0.43
20	79.74	-0.17	-0.08	0.33	0.56
22	80.93	0.02	0.07	0.35	0.51
24	81.89	0.12	0.15	0.34	0.45
26	82.75	0.08	0.10	0.24	0.31

Table S1. Difference between the oscillation amplitude θ_A^{ns} of a dipolar particle obtained by numerical simulation (ns) and the iterative approximate functions $\theta_A^{ap(n)}$ $(n = 1, ..., 4)$ for various field amplitudes $B_0^{\sim}$ (1st column), measured in $\left[\frac{\mu_0 m^2}{4\pi d^3}\right]$. The other field parameters are kept constant at $\omega = 0.05/(2\pi)$ and $B^{=} = 3\,\frac{\mu_0 m^2}{4\pi d^3}$ (Figure S2 a, b).

Table S1 shows that the numerical data points deviate from θ_A^{ap4} by less than 1°. Therefore, θ_A^{ap4} is a suitable approximate solution of θ_A^{ns}. The approximation θ_A^{ap4} derived here provides one possibility for a suitable functionality $\theta_A(B^{=}, B_0^{\sim}, \omega)$ as tested for the relevant value range of all field parameters.
